# Clinical evaluation of surgery for osteophyte-associated dysphagia using the functional outcome swallowing scale

**DOI:** 10.1371/journal.pone.0201559

**Published:** 2018-08-01

**Authors:** Mutsuya Shimizu, Tetsuya Kobayashi, Shizuo Jimbo, Issei Senoo, Hiroshi Ito

**Affiliations:** Department of Orthopaedic Surgery, Asahikawa Medical University, Asahikawa, Hokkaido, Japan; George Washington University, UNITED STATES

## Abstract

**Purpose:**

To investigate the surgical outcome of patients with osteophyte-associated dysphagia (OAD) using the functional outcome swallowing scale (FOSS).

**Methods:**

A retrospective chart review of 10 surgical cases of OAD (9 male and 1 female patient; mean age of 65 years) from 1982 to 2017 was performed, and radiographic evaluations were conducted by video fluoroscopic swallow study (VFSS) and conventional radiography. All OAD cases were treated at a single institution, and osteophytes were surgically resected by the anterior approach under gentle retraction of the affected esophagus. FOSS (0 for normal, 5 for worst) was used for clinical evaluations, and surgical complications were recorded.

**Results:**

VFSS evaluation of OAD showed that the affected osteophyte was located at C4/5 in four patients, followed by C3/4 in three patients. The mean FOSS showed significant improvement from 2.5 preoperatively to 0.3 postoperatively, and no major surgical complications were recorded. Comorbidities were diabetes mellitus in four patients, ossification of the posterior longitudinal ligament in three patients, and lumbar spinal stenosis (LSS) in three patients.

**Conclusion:**

Surgical treatment of OAD was promising, and all patients showed clinical recovery. Evaluation of dysphagia using FOSS was easy and reliable for OAD management, and FOSS 2 might be a good indication for surgical intervention.

## Introduction

Anterior cervical osteophytes are a common finding in degenerative spinal conditions, especially among the elderly population, with a reported 30% incidence of osteophytosis and with diffuse idiopathic skeletal hyperostosis (DISH) or Forestier disease [1–5].

Clinical studies have showed that up to 6% of patients with DISH manifested symptoms of osteophyte-associated dysphagia (OAD) [5–6]. However, clinical assessment of dysphagia has been heterogeneous among spine surgeons. Recent studies used the Functional Outcome Swallowing Scale (FOSS) (0 for normal, 1 for episodic dysphagia, 2 for dysphagia with no body weight (BW) change, 3 for BW loss <10%, 4 for BW loss >10%, and 5 for non-oral feeding) and reported reliable evaluation of dysphagia among patients with OAD (Table 1) [7]. The purpose of this study was to investigate the clinical features and surgical outcomes of patients with OAD.

**Table 1:** Functional outcome swallowing scale.

Stage	Symptoms
0	Normal function and asymptomatic
1	Normal function with episodic or daily symptoms of dysphagia
2	Compensated abnormal function, prolonged mealtime, no body weight loss
3	Decompensated abnormal function, body weight loss<10% over 6 months
4	Severely decompensated abnormal function, body weight loss>10% over 6 months
5	Non-oral feeding for all nutrition

## Methods

This study was a retrospective chart review of 10 consecutive surgical cases of OAD (male, 9; female, 1; mean age, 65.0 (55–78) years) treated at our institution between 1982 and 2017. The mean follow-up period was 4 years.

Radiographic evaluations were done using video fluoroscopic swallow study (VFSS) and conventional radiography. VFSS was performed at designated division of our hospital using continuous recording of esophageal movement while swallowing contrast medium.

Patients’ demographics, comorbidity, surgical complications were investigated, and clinical evaluation of dysphagia was performed using FOSS.

All patients gave written informed consent, and the study was approved by the institutional review board at our university.

## Surgical procedure

Under general anesthesia, the anterior Smith-Robinson type approach, usually from the left side, was used, and osteophytes were exposed with careful retraction of the affected esophagus. A high-speed diamond-tipped burr was used, and the affected osteophyte was thinned off to the original depth of the anterior vertebral cortex and width of Luschka’s joints. Intraoperative radiography was performed to confirm that the affected osteophytes were resected, and surfaces were smoothed as planned. Bone wax was applied to the surface of the resected area for hemostasis, although no additional procedures or spinal fusions were applied in all cases. The wound was cleansed and closed with soft drainage tube placed at the resected surface, mostly until next day postoperatively.

## Statistical analysis

We used t-tests to compare preoperative and postoperative FOSS in the same group. p-value of less than 0.05 was considered statistically significant. All statistical analyses were carried out using the StatView software (Abacus Concepts, Inc., Berkley, CA).

## Results

Radiographic analysis showed that maximal osteophyte formation was at C4/5 in four patients, C3/4 in three patients, C5/6 in two patients, and C6/7 in one patient.

VFSS analysis showed that the contrast medium was obstructed at the maximal osteophyte level in all patients.

The preoperative mean FOSS score was 2.5, and postoperative mean FOSS score was 0.3. Improvement of FOSS after surgery was significant in all patients (p<0.05).

Complications included transient recurrent nerve paralysis in one patient immediately postoperative, which recovered fully during follow-up. No major complication, such as laryngeal or esophageal perforation or vascular injury, was observed in our series. Recurrence of OAD was found in one patient after 9 years.

Patients’ comorbidities at enrollment included diabetes mellitus (DM, n = 4), ossification of the posterior longitudinal ligament (OPLL, n = 3), and lumbar spinal stenosis (LSS, n = 3) (Table 2).

**Table 2:** Clinical and demographic data and preoperative/postoperative FOSS scores.

No	Age	Sex	Comorbidity	Maximal neophyte level	Preop FOSS	Postop FOSS	P-value
1	55	M	LSS	C4/5	2	0	
2	68	F		C3/4	2	0	
3	61	M		C4/5	2	0	
4	56	M	OPLL LSS	C5/6	3	1	
5	71	M	LSS DM	C3/4	2	1	
6	57	M		C3/4	4	0	
7	67	M	OPLL DM	C4/5	2	1	
8	73	M	DM	C4/5	3	0	
9	59	M	OPLL DM	C5/6	2	0	
10	78	M	M	C6/7	3	0	
Average					2.5	0.3	< .001

DM, diabetes mellitus; FOSS, functional outcome swallowing scale; LSS, lumbar spinal stenosis; OPLL, ossification of the posterior longitudinal ligament

### Case presentation (patient no. 4)

**Fig 1:**
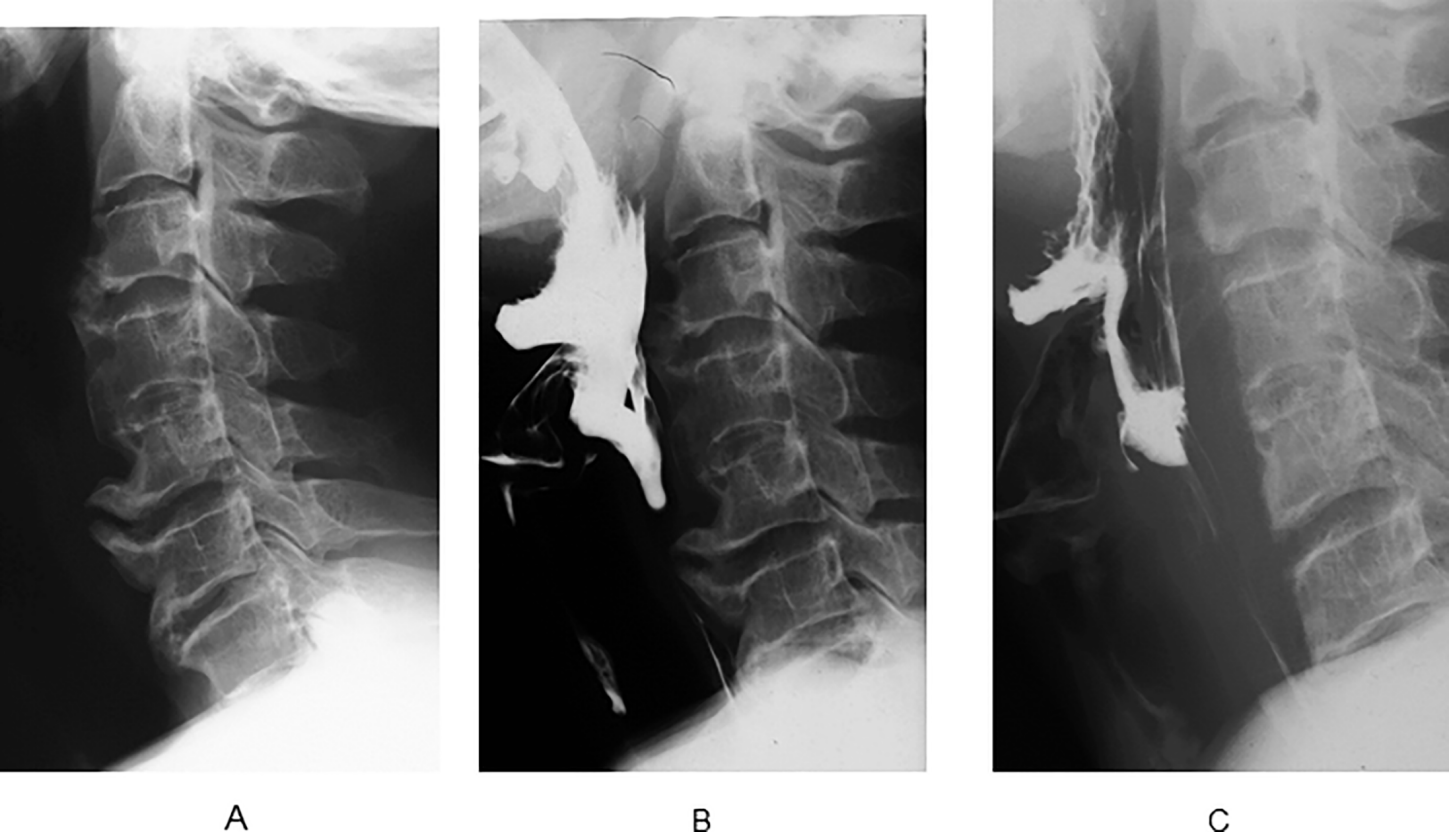
Preoperative and postoperative lateral radiographs of patient no. 4. (A) Cervical osteophytes at C5/6 and OPLL at C2/3. (B) Preoperative VFSS showed that the contrast medium was obstructed at C5/6 level and the esophagus was compressed. (C) Postoperative VFSS contrast medium was not obstructed, and the osteophyte at C5/6 was excised completely.

**Fig 2:**
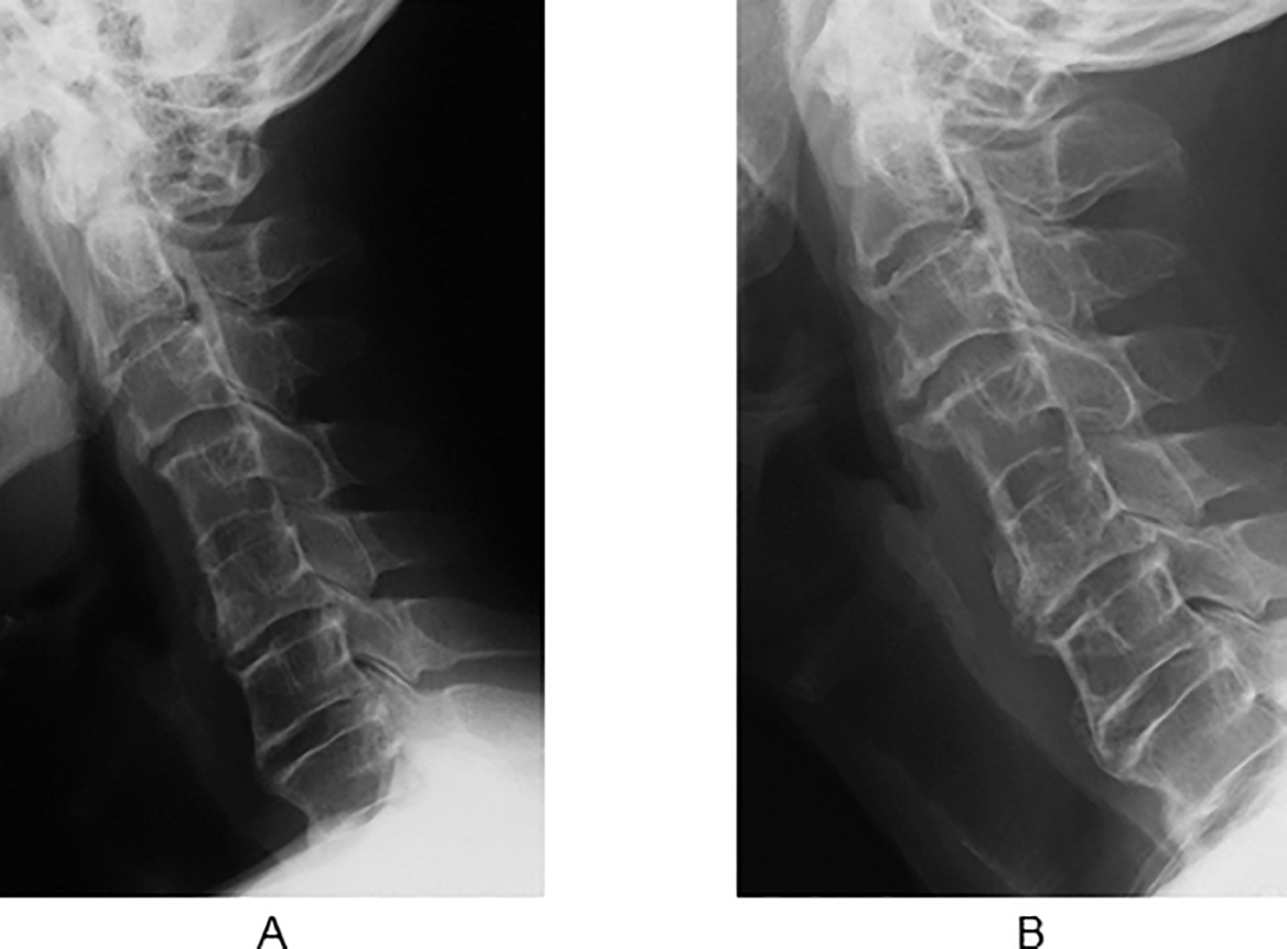
Lateral radiographs of patient no. 4 obtained 9 and 17 years after surgery (long-term follow-up). (A) Nine years after surgery, recurrent anterior osteophytes were observed at the C3/4 and C5/6 levels. (B) Seventeen years after surgery, the osteophytes were gradually enlarged at the C3/4 and C5/6 levels.

This case involved a 59-year-old male patient with OAD that started 6 years ago. The patient also showed DISH and OPLL with tendency of diffuse ligament ossification. Conservative treatments were unsuccessful, and surgical treatment was recommended. Preoperative VFSS revealed obstruction at the C5/6 osteophyte level. Postoperative VFSS showed normal movement of the esophagus, and reduced symptoms of dysphagia (FOSS 3 to 1) (Fig 1). Radiographic follow-up showed osteophytes at the C3/4 and C5/6 levels that demonstrated gradual regrowth at 9 and 17 years postoperatively (Fig 2).

## Discussion

This study showed that surgical treatment for OAD is promising and that all patients showed clinical recovery according to the FOSS scores.

In previous studies, patients with OAD had a higher prevalence of DM and OPLL [8,9]. Our series also confirmed the tendency for DM in 40% and OPLL in 30% of the cases. Denko reported that type 2 DM was a risk factor for DISH, and several growth factors and inflammatory mediators lead to proliferation of osteoblast [10,11].

In general, ossification of the anterior longitudinal ligament (OALL) is frequently associated with OPLL, and Song reported that OPLL and OALL coexisted in 11 of 17 patients [12].

We did not have information on the association with LSS. Interestingly, the scar tissue was changed to ossification after decompression surgery for LSS, and recurrence of stenosis induced reoperation surgery [13].

The affected site of the osteophyte was mainly at C3/5 according to a systematic review by Verlaan [8]. Their results coincided with our results. Degenerative changes are most frequent at these cervical levels, which might have been related to anterior osteophytosis and OAD [12].

Initial treatment strategies for OAD should be conservative, including diet modifications, postural changes, muscle relaxants, anti-reflux medications, and steroids. If conservative treatment is not effective, surgical treatment should be indicated for improving the patient’s quality of life (QOL) [2–5,9].

Oppenlander reported clinical improvement of dysphagia after surgical resection of the osteophyte in nine OAD cases [14].

Urrutia and Bono reported that dysphagia resolved within 2 weeks postoperatively, with no recurrence at approximately 5 years of follow-up of five OAD cases [15].

The present study found that the surgical result was satisfactory, but osteophyte regrowth at the site of resection was observed in one patient after 9 years of follow-up.

Miyamoto reported a long-term follow-up of seven patients with OAD and reported that two patients had recurrence of OAD symptom after 10 and 11 years, respectively, with one patient requiring reoperation. They found osteophyte regrowth in all patients, with an average of 1 mm per year, and recommended more than 10 years of follow-up for OAD [16].

Several assessment tools for dysphagia have been reported, including the Bazas dysphagia scoring system [17], the swallowing quality of life, and the dysphagia disability index [18]. Recently, the FOSS was reported by Salassa, which should be suitable for patients with OAD [3,4,7,19]. Salassa and Ozgursoy reported 13 cases of OAD evaluation using FOSS, and the preoperative FOSS was 2.4 on average, which improved to 1.0 postoperatively [19]. Their results showed that most surgical cases acquired normal function at 6-month follow-up. Erdur reported on a series of eight patients with OAD who recovered, with an improvement in the FOSS score from 2.6 preoperatively to 0.4 postoperatively [3]. Similarly, Vodičar reported nine OAD cases managed by surgical resection of cervical osteophytes, and the FOSS of these patient improved from 3.4 to 0.8 [4] (Table 3).

**Table 3:** Comparison of functional outcome swallowing scale (FOSS) scores between previous studies and our study.

Study	Cases (n)	Preop FOSS	Postop FOSS	Change in FOSS
Our study	10	2.5 [2–4]	0.3 [0–1]	2.2 [1–4]
Ozgursoy (2010)	13	2.4 [2–4]	1.0 [0–3]	1.4 [1–2]
Vodičar (2016)	8	3.4 [2–5]	0.8 [0–3]	2.6 [0–4]
Erdur (2017)	8	2.6 [2–3]	0.4 [0–1]	2.2 [1–3]

Our study and three previous reports showed almost the same degree of improvement in FOSS scores, and the surgical indication has been suggested according to these results. An FOSS score of 2, which shows compensated abnormal function manifested by significant dietary modifications or prolonged meal time, might be a good indication for surgical intervention, yielding good surgical results for OAD.

This study has some limitations. The incidence of OAD is low, as there were only 10 cases identified in 35 years at our university and 13 cases in 10 years at the Mayo Clinic. Therefore, these case reports provide no firm evidence, and further studies could provide sound guidelines for OAD treatment. FOSS is a useful tool. However, it is a physician-based evaluation and lacks patients’ perspectives of the quality of life or psychosocial aspects.

In conclusion, clinical and radiological outcomes of 10 OAD cases were studied. All patients recovered normal function. Considering other studies, surgery for OAD was found to be promising when indicated on the basis of an FOSS score of 2. Furthermore, long-term follow-up is important for recurrent dysphagia because of osteophyte regrowth.
